# Gene Delivery to Adipose Tissue Using Transcriptionally Targeted rAAV8 Vectors

**DOI:** 10.1371/journal.pone.0116288

**Published:** 2014-12-31

**Authors:** Silke Uhrig-Schmidt, Matthias Geiger, Gerd Luippold, Gerald Birk, Detlev Mennerich, Heike Neubauer, Dirk Grimm, Christian Wolfrum, Sebastian Kreuz

**Affiliations:** 1 Boehringer Ingelheim Pharma GmbH & Co. KG, Biberach an der Riss, Germany; 2 Swiss Federal Institute of Technology, ETH Zurich, SLA C92, Institute of Food Nutrition and Health, Schwerzenbach, Switzerland; 3 Centre for Infectious Diseases/Virology, Heidelberg University Hospital and Cluster of Excellence CellNetworks, Heidelberg University, Im Neuenheimer Feld 267, Heidelberg, Germany; University of North Carolina at Chapel Hill, United States of America

## Abstract

In recent years, the increasing prevalence of obesity and obesity-related co-morbidities fostered intensive research in the field of adipose tissue biology. To further unravel molecular mechanisms of adipose tissue function, genetic tools enabling functional studies *in vitro* and *in vivo* are essential. While the use of transgenic animals is well established, attempts using viral and non-viral vectors to genetically modify adipocytes *in vivo* are rare. Therefore, we here characterized recombinant Adeno-associated virus (rAAV) vectors regarding their potency as gene transfer vehicles for adipose tissue. Our results demonstrate that a single dose of systemically applied rAAV8-CMV-eGFP can give rise to remarkable transgene expression in murine adipose tissues. Upon transcriptional targeting of the rAAV8 vector to adipocytes using a 2.2 kb fragment of the murine adiponectin (mAP2.2) promoter, eGFP expression was significantly decreased in off-target tissues while efficient transduction was maintained in subcutaneous and visceral fat depots. Moreover, rAAV8-mAP2.2-mediated expression of perilipin A – a lipid-droplet-associated protein – resulted in significant changes in metabolic parameters only three weeks post vector administration. Taken together, our findings indicate that rAAV vector technology is applicable as a flexible tool to genetically modify adipocytes for functional proof-of-concept studies and the assessment of putative therapeutic targets *in vivo*.

## Introduction

Obesity is a leading preventable cause of death worldwide with increasing incidence in adults and children, and authorities view it as one of the most serious public health problems of the 21st century [1]. Today, adipose tissue is no longer seen as an inert depot for lipid storage, but as a metabolically active, plastic, endocrine tissue [2]. There are two major, functionally different types of adipose tissues in mammals: white and brown. White adipose tissue (WAT) is highly adapted to store excess energy in the form of triglycerides inside lipid droplets, which are – besides other mechanisms – coated and regulated by members of the perilipin family of proteins. Under basal conditions, perilipins act by restricting the access of cytosolic lipases to lipid droplets and thus promote triacylglycerol storage [3]. In contrast to WAT, brown adipose tissue (BAT) oxidizes chemical energy to produce heat as a defense against hypothermia and obesity. Brown fat-like cells are found scattered within WAT of rodents and humans [4]. The development and presence of these brown in white (brite) cells – a process called browning – has recently been shown to be associated with improved metabolic phenotypes [5], [6]. Understanding the underlying mechanisms of browning and other processes that activate BAT is crucial for a therapeutic benefit which is the reduction of weight and plasma lipid levels in obese and hyperlipidemic patients. To study metabolic processes, genetic engineering of adipose tissue is essential but this, however, currently mostly relies on the time-consuming generation of transgenic animals [7].

Thus far, only very few studies have tackled viral vector-mediated transduction of adipose tissue and cells which reside therein (reviewed in [2], [8], [9]). Amongst the popular panel of viral vectors for *in vivo* gene transfer, very promising candidates are based on adeno-associated viruses (AAV) due to their overall good safety profile, apathogenicity and low immunogenicity [10]. AAVs belong to the family of *Parvoviridae* and the genus Dependovirus. This classification is based on their requirement for co-infection with a helper virus (e.g., adenoviruses (Ad) or herpes simplex viruses (HSV)) to complete their life cycle [11]. To date, 14 serotypes and multiple variants have been described, which differ in primary sequence, capsid structure, antigenic diversity and tissue tropism [12]. In contrast to liver and skeletal muscle, which are well established target organs for AAV-mediated gene transfer, adipose tissue was selected as a target for AAV transduction in only three studies [8], [9], [13].

Based on the small amount of available data and the need for a flexible tool to genetically engineer adipose tissue, we here tested various AAV serotypes regarding their efficacy in transducing different fat depots following local and systemic application *in vivo*. Our study revealed AAV8 to most efficiently transduce adipose tissue after local injection into visceral fat pads of mice. Systemically applied, the broad transgene expression profile of rAAV8 with cytomegalovirus promoter (CMV)-mediated expression of the enhanced green-fluorescent protein (eGFP) was almost completely restricted to adipose tissues when a minimal murine adiponectin promoter (mAP2.2) was used instead of CMV. To exemplify an application of the transcriptional targeting approach, we overexpressed Perilipin A (PlinA) from mAP2.2 and – strikingly – observed significant changes in metabolic parameters in rAAV8-mAP2.2-PlinA-treated animals only three weeks following vector application. These findings highlight the potential of transcriptionally targeted rAAV8 vectors as a versatile alternative to the laborious generation of transgenic animal models for the study of adipocyte biology and the assessment of putative therapeutic targets.

## Results

### AAV8 is a superior serotype for transduction of adipose tissue

**Figure 1:**
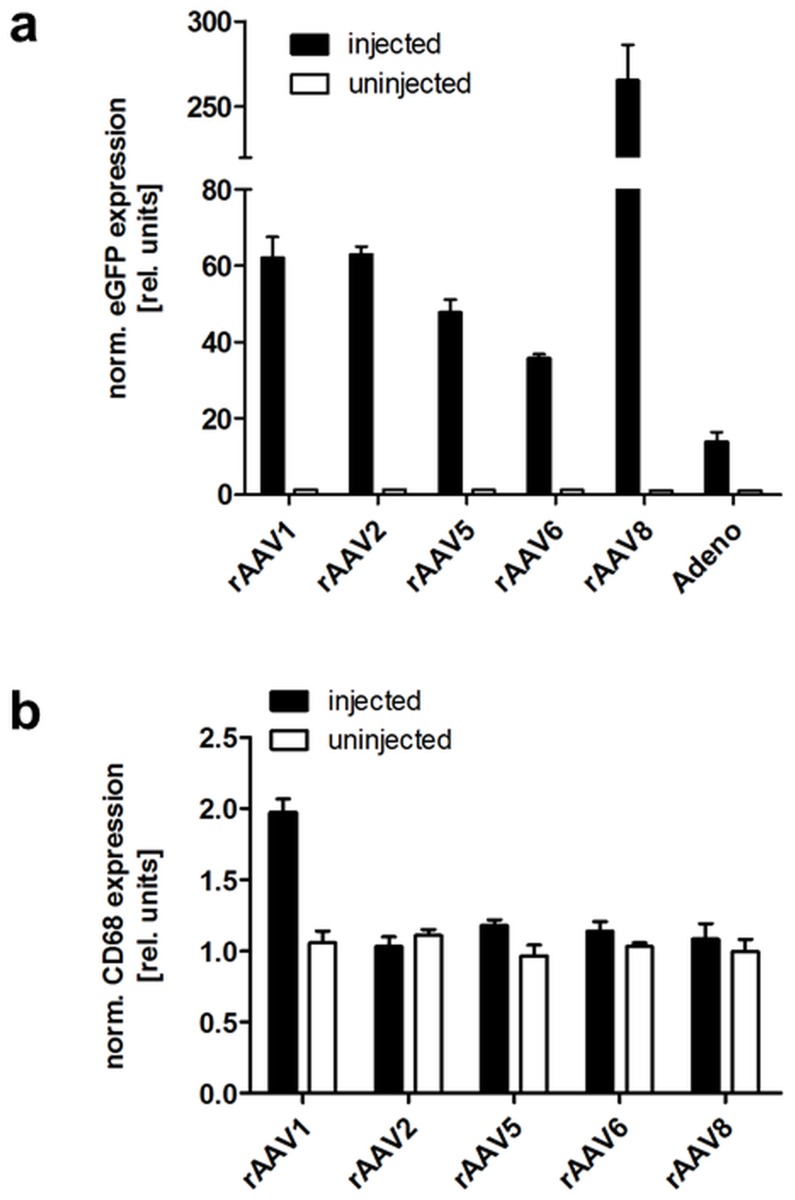
Local application of rAAV serotypes 1, 2, 5, 6 and 8 and rAd into visceral fat pads. C57BL/6 mice were injected with 4.2×10^10^ VG per fat pad of rAAV-CMV-eGFP vectors or rAd-CMV-eGFP, respectively. Four weeks post-injection, mice were sacrificed and eGFP (a) or CD68 expression (b) was determined in cDNA from injected (black bars) and non-injected (white bars, not visible in panel a) fat pads by qPCR analysis. Normalization to the housekeeping gene 36B4 was performed and relative gene expression is depicted in the graph. Values indicate the mean of three animals, error bars show SEM.

On the basis of a previous study by Mizukami and colleagues [13] in which AAV1 was reported to transduce adipose tissue significantly more efficiently than AAV serotype 2 through 5 following local application, we first compared transduction efficacy of several AAV serotypes including AAV1 as well as Ad5 after direct injection into visceral fat pads of mice. AAV serotypes 1, 2, 5, 6 and 8 were produced as recombinant vectors encoding enhanced green-fluorescent protein (eGFP) controlled by the cytomegalovirus (CMV) promoter in a single-stranded vector genome conformation. The expression cassette was flanked by serotype 2 packaging signals (ITRs), thus, rAAV1, 5, 6 and 8 were produced as pseudotypes [14]. Recombinant AAV vectors and rAd were administered at a dose of 4.2×10^10^ vector genomes (VG) per fat pad of 8 weeks old male C57BL/6 mice (n = 6). Animals were sacrificed 4 weeks post-injection and fat pads as well as other organs, i.e. liver, spleen, colon and small intestine, were isolated. Quantitative real-time PCR (qPCR) revealed that rAAV8 clearly outperformed all other rAAVs tested and rAd with respect to eGFP expression in the injected fat pad (Fig. 1a). Moreover, except for rAAV1 in the liver and rAAV6 in the small intestine, transgene expression was absent in all other organs that were analyzed (S1 Fig.).

Although AAV is known to induce only mild cellular immune responses [15], we nevertheless checked for tissue inflammation by assessing macrophage infiltration in rAAV-injected vs. non-injected fat pads. With the exception of rAAV1, which showed two-fold higher expression of the macrophage marker CD68 in the injected fat pad compared to the uninjected control, qPCR analysis confirmed equal CD68 expression levels in both fat pads for all other rAAV vectors (Fig. 1b).

### Minimal adiponectin promoter restricts rAAV8-mediated gene expression to adipose tissue following systemic application

Having demonstrated that AAV8 is best suited to transduce the murine visceral fat upon local application, we aimed to explore transgene expression in our organ of interest also after systemic vector administration. Due to the broad tissue tropism of rAAV8 vectors upon systemic application in rodents [16], we decided to apply a transcriptional targeting strategy to restrict transgene expression to the target tissue. Based on an earlier publication by Koshiishi and co-workers [17] and with regard to the limited packaging capacity of rAAV vectors [18], we selected a 2.2 kb minimal murine adiponectin promoter (mAP2.2, S2 Fig.) for adipose-tissue specific expression and cloned this fragment in front of an *egfp* cDNA. To assess functionality of this construct, we electroporated murine 3T3L1 pre-adipocytes and subsequently differentiated the electroporated cells into adipocytes as previously described elsewhere [17]. QPCR analysis demonstrated greatly increasing eGFP expression in the course of differentiation – in parallel with endogenous adiponectin expression – whereas eGFP expression was notably lower in undifferentiated controls (S3 Fig.). Successful differentiation into adipocytes was proven by oil red O staining (S3 Fig.).

**Figure 2:**
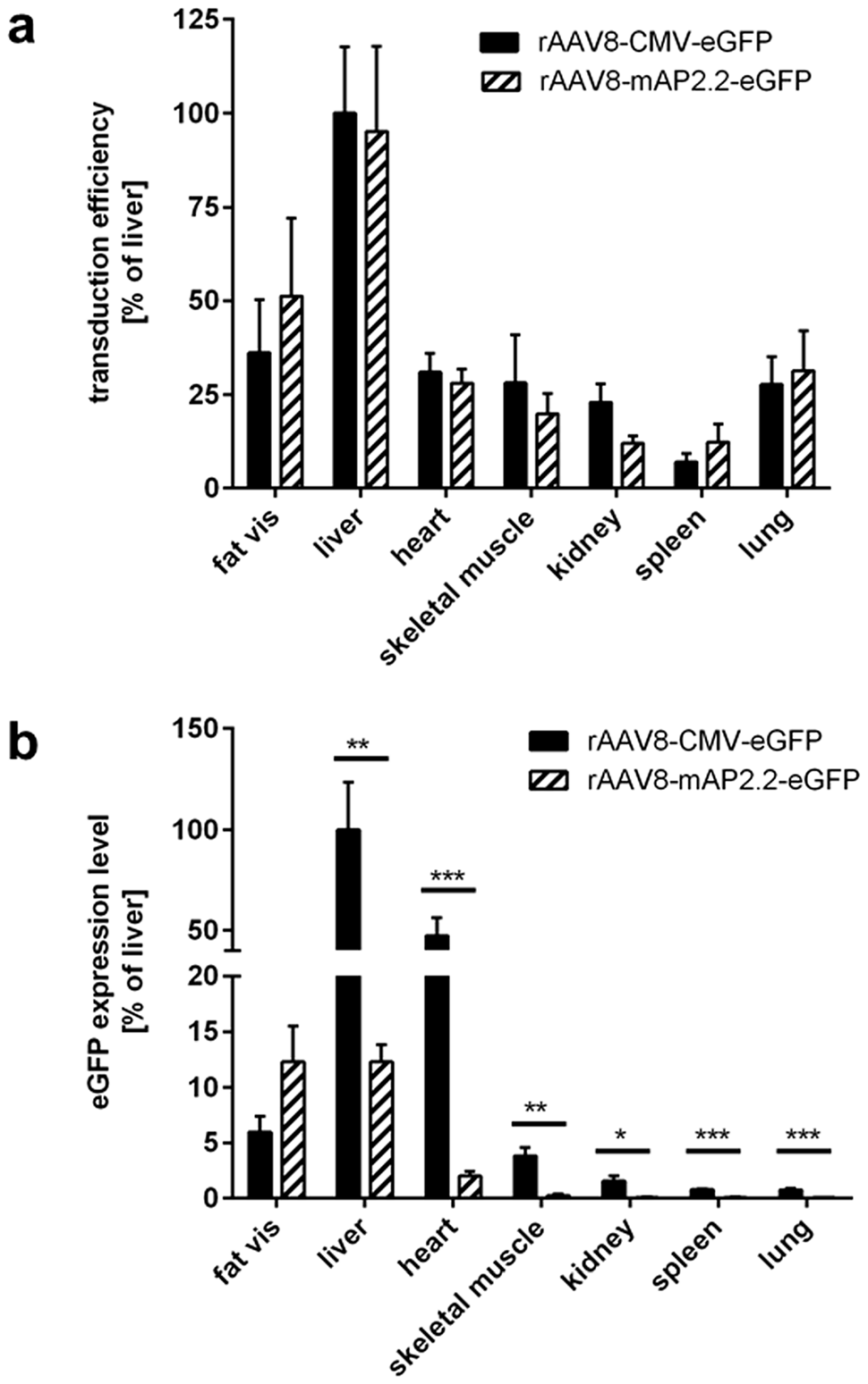
Systemic application of rAAV8-CMV-eGFP and rAAV8-mAP2.2-eGFP. C57BL/6 mice were injected with 10^12^ VG per animal of rAAV vectors (rAAV8-CMV-eGFP: black bars, rAAV8-mAP2.2-eGFP: hatched bars) or PBS (not shown) as a control via the tail vein. Three weeks post-application, mice were sacrificed and tissue samples from various organs were taken. (a) Biodistribution of vector genomes indicating transduction efficiency was assessed by measuring eGFP copies per ng total DNA in the different organs via qPCR. The mean value obtained for liver samples of the rAAV8-CMV-eGFP group (9.4×10^3^ copies per ng total liver DNA) was set to 100%. (b) EGFP expression levels were determined by qPCR and normalized to the housekeeping gene RNA-pol-II. The mean of the target/reference ratio calculated for liver samples of the rAAV8-CMV-eGFP group was set to 100%. Values indicate the mean of five animals, error bars show SEM ***P<0.001; **P<0.01 and *P<0.05.

Next, we compared the capability of the two promoters to transcriptionally target rAAV8-mediated transgene expression to adipose tissue *in vivo*. To this end, rAAV8-mAP2.2-eGFP was packaged in single-stranded genome conformation and applied at a dose of 10^12^ VG per animal via the tail vein of C57BL/6 mice (n = 5). Recombinant AAV8-CMV-eGFP was administered at the same vector dose as a control with ubiquitous transgene expression; moreover, PBS was used as mock control. Animals were sacrificed three weeks post AAV injection and various organs, including fat, liver, heart, skeletal muscle, kidney, spleen and lung, were isolated. QPCR was carried out to compare the number of VG in the aforementioned organs relative to the value obtained in liver samples of the rAAV8-CMV-eGFP group, which was set to 100% (corresponding to 9.4×10^3^ copies per ng total liver DNA). As expected, both vectors showed a similar biodistribution owing to the AAV8 capsid which they have in common (Fig. 2a). Consistent with previous reports [19], [20], the highest number of rAAV8 VG was detected in liver samples. Interestingly, visceral fat ranked behind liver but higher than heart and skeletal muscle, two organs previously described as excellent AAV8 targets. Moreover, a notable amount of VG was observed in kidney, spleen and lung, whereas in mock-treated animals, VG signals were at background level.

With respect to transgene expression, rAAV8-CMV-eGFP exceeded its transcriptionally targeted counterpart rAAV8-mAP2.2-eGFP in all organs except for visceral fat (Fig. 2b). In fact, transcriptional activity in adipose tissue was slightly higher in case of the mAP2.2 promoter compared to CMV, whereas eGFP expression was significantly diminished in liver (−87.7%), heart (−95.8%) and skeletal muscle (−93.3%) and almost completely abolished in kidney, spleen and lung of rAAV8-mAP2.2-eGFP-treated animals compared to animals injected with rAAV8-CMV-eGFP.

**Figure 3:**
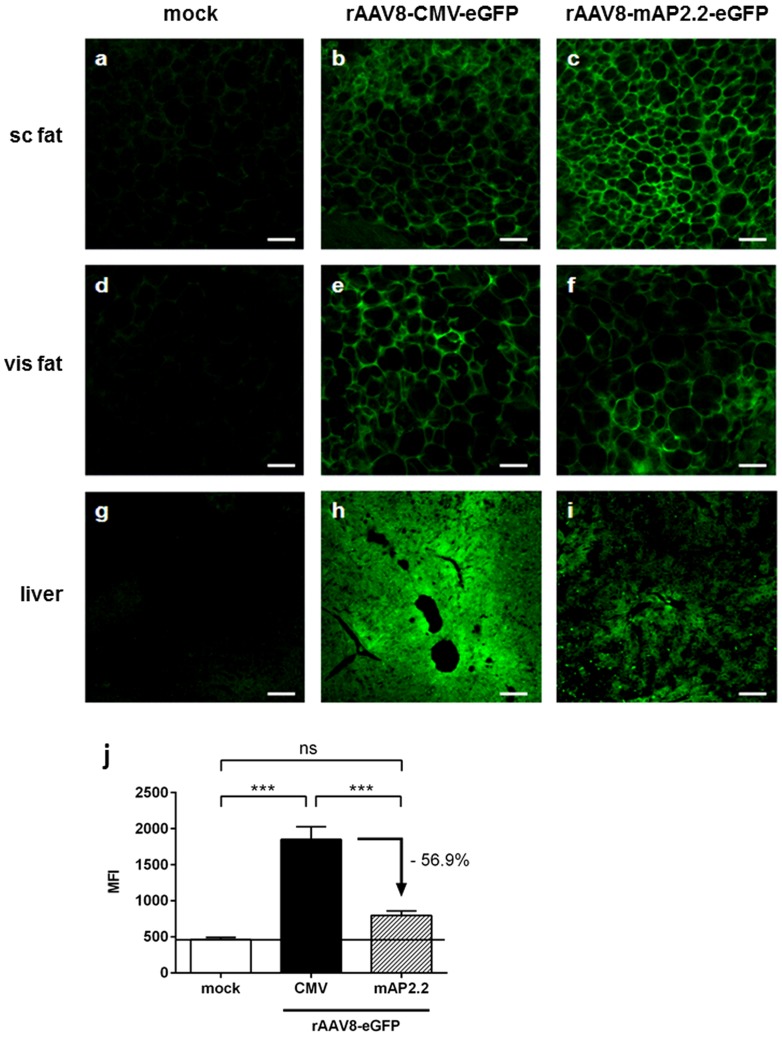
Histological assessment of eGFP expression following systemic application of rAAV8-CMV-eGFP and rAAV8-mAP2.2-eGFP. Five C57BL/6 mice per group were injected with 10^12^ VG per animal of rAAV8-CMV-eGFP, rAAV8-mAP2.2-eGFP or PBS as a mock control via the tail vein. Three weeks post-injection, mice were sacrificed and tissue samples from subcutaneous (sc) fat, visceral (vis) fat and liver were taken and rapidly frozen in liquid nitrogen. Cryosections of liver and adipose tissues were evaluated for eGFP expression by confocal fluorescence microscopy using direct imaging of tissue slices. (a–i) Representative images are shown for sc fat (a–c), vis fat (d–f) and liver (g–i). (a,d,g: mock; b,e,h: rAAV8-CMV-eGFP; c,f,i: rAAV8-mAP2.2-eGFP), scale bar: 50 µm. (j) EGFP expression (mean fluorescence intensity (MFI)) in the liver was quantified using the “Histo” function of the ZEN software. The horizontal line indicates background fluorescence detected in mock-treated samples. Values represent the mean of 25 random, individual visual fields per group (five individual sections per animal), error bars show SEM. ***P<0.001; ns: not significant.

To visualize the extent of eGFP expression on the protein level, tissue cryo-sections of liver, visceral and subcutaneous fat of all animals were directly analyzed by confocal microscopy. Histological analyses revealed similar eGFP levels in adipocytes of visceral and subcutaneous fat in rAAV8-CMV-eGFP- and rAAV8-mAP2.2-eGFP-treated mice (Fig. 3a–f), thus confirming the results obtained by qPCR analysis (Fig. 2b). In the liver, a strong eGFP signal was observed in animals transduced with rAAV8-CMV-eGFP (Fig. 3h), whereas in animals that received rAAV8-mAP2.2-eGFP, eGFP fluorescence was distinctly lower (Fig. 3i). To investigate whether this difference was statistically significant, we quantified the mean fluorescence intensity (MFI) in five independent sections per animal and randomly picked one visual field per section for the analysis. Results of the quantification (Fig. 3j) showed a significant reduction of eGFP fluorescence in rAAV8-mAP2.2-eGFP-treated vs. rAAV8-CMV-eGFP-treated animals. Interestingly, the transcriptionally targeted rAAV8 vector reached only slightly higher eGFP expression levels in the liver than the unspecific background fluorescence detected in mock control samples, thus its activity was markedly reduced (−56.9%) compared to the strong eGFP signal originating from CMV promoter-driven liver expression. Although we cannot rule out residual liver expression, we nevertheless were prompted by the strong rAAV8-mAP2.2-mediated transgene expression in adipose tissues to pursue a functional study using this construct.

### Systemic administration of rAAV8-mAP2.2-PlinA results in robust Perilipin A expression in visceral and subcutaneous fat depots and changes in metabolic parameters of mice

**Figure 4:**
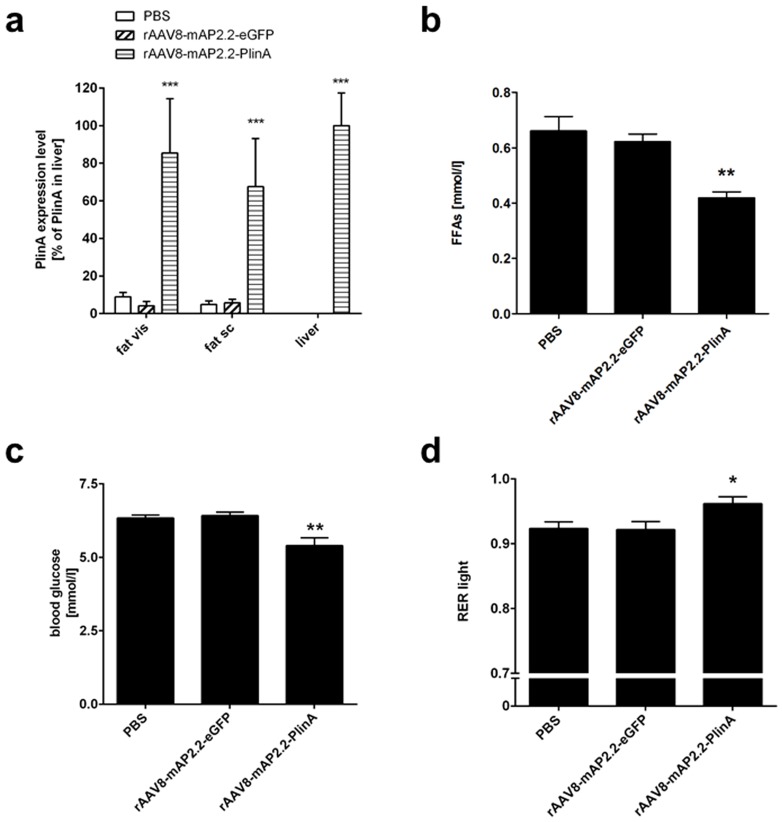
Systemic application of rAAV8-mAP2.2-eGFP and rAAV8-mAP2.2-PlinA. C57BL/6 mice were injected with 10^12^ VG per animal of rAAV vectors (rAAV8-mAP2.2-eGFP: hatched bars, rAAV8-mAP2.2-PlinA: striped bars) or PBS (white bars) as a mock control via the tail vein. Three weeks post application, mice were sacrificed and tissue samples from subcutaneous (sc) fat, visceral (vis) fat depots and liver were taken. (a) Transgenic PlinA expression levels were determined by qPCR. Gene expression data were normalized to RNA-pol-II and the mean of the target/reference ratio calculated for liver samples of the rAAV8-mAP2.2-PlinA group was set to 100%. (b–c) Serum levels of free fatty acids (b) and blood glucose (c). (d) Respiratory exchange ratio (RER): results are illustrated within the physiologically relevant window of 0.7 to 1.0. Values indicate the mean of at least three animals, error bars show SEM. ***P<0.001; **P<0.01 and *P<0.05.

We next investigated whether rAAV8-mAP2.2-mediated expression of the lipid-droplet associated protein Perilipin A (PlinA) in adipocytes would give rise to a metabolic phenotype in rAAV-treated animals. Therefore, a codon-optimized version of murine PlinA cDNA was cloned downstream of the already described mAP2.2 promoter. Recombinant AAV8-mAP2.2-PlinA vectors were generated as described before and administered intravenously at a dose of 10^12^ VG per animal (n = 5). Recombinant AAV8-mAP2.2-eGFP and PBS were used as controls. Animals were euthanized three weeks post rAAV application and fat depots (subcutaneous (sc) and visceral (vis)) and the liver as the major off-target organ were isolated. As before, the number of VG was determined by qPCR and again, liver was significantly better transduced by rAAV8 than visceral and subcutaneous fat (S4 Fig.). Moreover, qPCR analysis of the individual fat depots and liver showed comparable expression levels of transgenic PlinA in the rAAV8-PlinA-treated animals, whereas transgenic PlinA transcription was at background level in adipose tissues of rAAV8-eGFP- and mock-treated animals and absent in liver (Fig. 4a). Next, we assessed free fatty acids (FFA), blood glucose and the respiratory exchange ratio (RER), the key metabolic parameters that may be affected by PlinA overexpression and that have previously been described to be altered in perilipin A knock-out mice [21]. As depicted in Fig. 4b serum free fatty acid levels were significantly reduced in rAAV8-PlinA-treated animals and moreover, we also detected significantly lower blood glucose levels in the rAAV8-PlinA group compared to control animals (Fig. 4c). To further validate these two findings, we analyzed the RER by using metabolic cages. By definition, an RER of 0.7 indicates that fat is the predominant fuel source. In contrast, a value of 1.00 or greater is indicative of carbohydrate being predominantly metabolized. We measured an average RER of 0.92 for both mock-treated and rAAV8-eGFP-control animals, respectively, and a significantly higher RER of 0.96 in rAAV8-PlinA-treated animals (Fig. 4d). In line with our predictions, the three metabolic parameters analyzed consistently indicate a more pronounced use of glucose as energy source in rAAV8-PlinA-treated mice compared to animals with normal, endogenous PlinA expression levels.

## Discussion

The numerous functions of adipose tissue in different diseases such as obesity, type 2 diabetes and cardiovascular complications have become the subject of intensive research. Recently, major advances have been achieved in this field including the discovery of brown adipose tissue in humans [22]–[26]. For the further investigation of molecular mechanisms and pathways underlying the complex biology of adipose tissue, access to tailored genetic tools is essential. However, while the use of transgenic animal models is well established, examples describing viral vectors as tools to target adipose tissue remain rare [2], [8], [9], [13]. In an attempt to identify a viral vector platform allowing efficient and selective gene delivery to different adipose tissue depots in mice, we here investigated the potential of recombinant AAV vectors.

By comparing the transduction efficiency of five eGFP-expressing rAAVs (AAV1, -2, -5, -6, -8) and an adenoviral vector (Ad) following local injection into the visceral fat pad of C57BL/6 mice, we identified rAAV8 as the most efficacious vector, yielding transgene expression levels that were up to 7-fold higher compared to the other tested AAV serotypes. However, contrary to the study by Jimenez and colleagues [8], who also identified rAAV8 as an efficient serotype after local injection into WAT fat pads, we did not observe substantial transduction of other organs following local rAAV8 application. Consistent with an earlier study by Mizukami and colleagues [13], we confirmed robust transgene expression for rAAV1, which was similar to rAAV2-mediated eGFP expression and notably higher than the expression levels observed for rAAV5 and -6. Ad-mediated eGFP expression was least efficient in our experiment. Noteworthy, the Ad vector was not produced and titrated by us but was purchased at BioFocus, so the side-by-side comparison of rAAV and rAd might not be truly comprehensive. However, a likely explanation for the lower transduction of adipose tissue by Ad is the transient nature of Ad-mediated transgene expression, typically resulting from a strong initial inflammatory response and the subsequent elimination of transduced cells [27]–[29]. The applicability of Ad vectors in models where stable transgene expression is required could nevertheless be provided by high-capacity adenoviral vectors (HC-AdVs) which lack all viral coding sequences and were shown to mediate long-term transgene expression *in vivo* [30]. When investigating AAV vector-mediated inflammation, we observed upregulation of CD68 solely in fat pads injected with rAAV1 but no other AAV serotype, underlining the generally low immunogenic profile of rAAV vectors.

Although local fat pad injection is a safe and well-established procedure it is limited in throughput and by the fact that it results in locally restricted transgene expression at the site of injection. To overcome these limitations, we aimed to assess rAAV8-mediated gene delivery to adipose tissue by using a systemic application protocol. Due to the well-described tropism of rAAV8 vectors, with liver, heart and skeletal muscle being the main target organs [16], [19], we decided to apply a transcriptional targeting strategy in order to restrict transgene expression to our tissue of interest. Thus, in addition to a standard CMV-eGFP expression cassette, we generated a vector construct driving eGFP expression under the control of an adipocyte-specific promoter. For this purpose we used a 2.2 kb fragment of the murine adiponectin promoter (mAP2.2), which was previously described to contain the minimal regulatory elements required for specific transcriptional activity during adipocyte development, i.e. binding sites for members of the CCAAT/enhancer binding protein (C/EBP) transcription factor family [17]. Activity and selectivity of the mAP2.2-eGFP construct was confirmed *in vitro* in 3T3L1 pre-adipocytes in which increasing mAP2.2-driven eGFP expression was detected in parallel with endogenous adiponectin expression during the course of differentiation, whereas eGFP expression remained at a low level in transfected but undifferentiated controls. When packaged as rAAV8 vectors and injected systemically, both the ubiquitous CMV-eGFP and the adipocyte-specific mAP2.2-eGFP showed the highest transduction rates in the liver while notably lower but still robust transduction levels were observed in skeletal muscle and heart of mice. This transduction profile is well in line with literature [19], [20] but surprisingly, the transduction levels in visceral fat were only excelled by the levels observed in liver and were significantly higher compared to skeletal muscle, heart and the other organs investigated, thus underscoring the potential of rAAV8 vectors to target adipose tissues *in vivo*. Noteworthy, in comparison to local application, AAV was injected into the tail vein at a higher dose in order to account for the dissemination of the AAV vectors throughout the different tissues, probable renal clearance of a portion of the injected particles, and – as mentioned above – the known liver tropism of AAV8. Despite the robust liver transduction, when analyzing transgene expression levels, we observed a strong de-targeting from liver, heart and skeletal muscle in case of rAAV8-mAP2.2-eGFP, while expression in visceral fat was maintained. Liver cryo sections confirmed a significantly lower MFI in case of rAAV8-mAP2.2-eGFP- compared to rAAV8-CMV-eGFP-treated animals and - more importantly - only slightly higher MFI than the unspecific background fluorescence of mock-treated animals. With respect to adipose tissues, a very specific and strong fluorescence signal was detected in the adipocytes of rAAV8-CMV-eGFP- and rAAV8–mAP2.2-eGFP-treated mice, irrespective of whether it came from the subcutaneous or visceral depot, indicating that equally efficient transgene expression can be evoked in both types of fat following systemic rAAV8 application. The markedly reduced eGFP expression we observed in the liver when using the mAP2.2 promoter differs substantially from the recently published study by O'Neill and colleagues [9] in which the use of a 700 bp enhancer/promoter region of the human adiponectin promoter was insufficient to suppress eGFP expression in the liver. The two studies also differ conceptually as the approach pursued by O'Neill and colleagues required the additional incorporation of binding sites for the liver-specific miR-122 into the vector genome to achieve liver de-targeting. In the context of future therapeutic applications, it is well conceivable that a miRNA-based de-targeting strategy can result in adverse dysregulation of endogenous miR-122. In addition, a miRNA-based de-targeting strategy requires the incorporation of binding sites for each tissue in which off-targeting can occur, which complicates vector generation and restricts the size for a therapeutic transgene. Conversely, our promoter-based strategy circumvents these concerns as we neither interfere with endogenous miRNA levels nor depend on multiplexing of miRNA binding sites in our vector genome. In brief, we conclude from our thorough analysis that by using the mAP2.2 promoter instead of the CMV promoter, a substantial de-targeting from the rAAV8 main target organs can be achieved, while maintaining robust transgene expression in adipose tissues.

On the basis of the observed tissue selectivity of the mAP2.2 promoter, we performed a set of *in vivo* experiments to evaluate whether the rAAV8-mAP2.2-mediated expression of a functional gene of interest would be sufficient to induce phenotypic changes in an expected manner. For this purpose, we chose PlinA, a lipid droplet-associated protein that protects stored lipids from lipases and likewise has a role in regulating triacylglycerol hydrolysis as it was recently demonstrated by the characterization of PlinA null mice [3], [21], [31]. In our experiment, we observed robust PlinA expression levels in subcutaneous and visceral fat depots and – as expected from the previous experiment – also in the liver. With respect to metabolic parameters, we measured lower FFA levels in rAAV8-mAP2.2-PlinA-treated animals compared to the controls which could be attributed to the lipid-droplet-protective function of PlinA in adipocytes. Interestingly, peri^−/−^ mice are characterized by the opposite phenotype, i.e. elevated FFA levels [21]. A plausible explanation for our observation could be the inaccessibility of fatty acids for metabolism caused by enhanced protection of lipid droplets by elevated PlinA levels in rAAV8-mAP2.2-PlinA-treated mice. Consequently, if free fatty acids cannot be used as an energy source, carbohydrates are likely to be used. To test this hypothesis, we measured blood glucose in all three groups and indeed observed lower blood glucose levels in the PlinA group compared to controls. The evidence that PlinA overexpression could lead to a shift towards glucose as a fuel source owing to the capture of fatty acids inside lipid droplets was further validated by metabolic cage studies. In fact, we observed a higher RER in PlinA-overexpressing mice compared to controls. Briefly, all three metabolic analyses clearly indicate a shift in energy source towards carbohydrates in rAAV8-mAP2.2-PlinA-treated animals.

Taken together, we here provide evidence that transcriptionally targeted rAAV8 vectors can mediate robust transduction of different adipose tissue depots giving rise to transgene expression levels that are sufficient to perform functional studies *in vivo*, as exemplified with PlinA overexpression. Future directions to further improve the applicability of the system could include the use of additional promoters to enable specific targeting of different cell types within adipose tissue, e.g. pre-adipocytes or brown adipocytes. Moreover, as already described by others [8], [32]–[34], our system is compatible with the incorporation of binding sites for miR-122 to the 3′-UTR of the transgene expression cassette to further reduce residual expression in the liver. However, as discussed above, this approach bears the risk of interfering with endogenous miRNA levels which might cause adverse side-effects. Yet another possible future improvement is selection and use of an AAV capsid mutant with reduced liver tropism as compared to the hepatotropic AAV8 using molecular evolution technologies such as DNA family shuffling [35] or peptide display [36]. In summary, our data indicate that rAAV8 vectors in combination with a straight-forward transcriptional targeting approach are a versatile tool to study adipocyte biology and/or to analyze putative therapeutic targets *in vivo*.

## Materials and Methods

All animal experiments described in this study were conducted in accordance with National Health and Medical Research Committee Guidelines for Animal Experimentation. The protocols were approved by the Regional Commission Tubingen (permit number 35/9185.81-8) and the Veterinary Office Zurich.

### Plasmids

The construction of pFB-CMV-eGFP was described in [37] and the plasmid was kindly provided by Dr. Robert Kotin (NIH). pFB-mAP2.2-eGFP was cloned by replacing the CMV promoter in pFB-CMV-eGFP with the minimal adiponectin promoter fragment (mAP2.2) published by Koshiishi and colleagues [17], which was generated by gene synthesis (Life Technologies, Darmstadt, Germany). For the cloned sequence, see S2 Fig. The eGFP gene in pFB-mAP2.2-eGFP was replaced by a codon-optimized version of the perilipin A open reading frame (NM_001113471.1), which was also generated by gene synthesis (Life Technologies). The resulting pFB-mAP2.2-PlinA served as the vector plasmid for packaging rAAV vectors encoding mouse PlinA. pDP1rs, pDP2rs, pDP5rs, pDP6rs and pDP8.ape (PlasmidFactory, Bielefeld, Germany) encoding AAV2 *rep*, the respective AAV1, 2, 5, 6 or 8 *cap* and Ad5 helper functions [38], [39] were used for rAAV vector production.

### Production of rAAV vectors

All rAAV vectors were produced in HEK293-H (Invitrogen, Darmstadt, Germany) cells by the helper virus-free, two-plasmid-based production method [38], [39]. Briefly, subconfluent cells were co-transfected by the calcium phosphate method with equimolar amounts of a *rep*/*cap*/helper plasmid (pDP1rs, pDP2rs, pDP5rs, pDP6rs or pDP8.ape) and a vector plasmid (pFB-CMV-eGFP, pFB-mAP2.2-eGFP or pFB-mAP2.2-PlinA). At 72 h post-transfection, cells were lyzed, treated with benzonase and further purified by CsCl density gradient centrifugation [14] at 45,000 rpm for 20 h at 21°C. Fractions from the gradient were collected, peak fractions were pooled, dialyzed against 1× DPBS containing 10% glycerol, concentrated (Amicon Ultra-15, Millipore, Schwalbach, Germany), filter-sterilized and stored at −80°C. Genomic particle titers were determined by real-time qPCR (*TaqMan HT 7900, Applied Biosystems, Darmstadt, Germany*) using transgene- or promoter-specific primers and probes (S1 Table). All reactions were performed in triplicates and analyzed using SDS2.4 software (*Applied Biosystems*).

### Electroporation and differentiation of 3T3L1 cells

3T3L1 cells (ATCC number: CL-173) were maintained in Dulbecco's modified Eagle's medium (DMEM) (Lonza, Cologne, Germany) supplemented with 10% fetal calf serum (FCS) (Invitrogen, *Darmstadt*, Germany) in a humidified incubator with 5% CO_2_ at 37°C. Prior to transfection, cells were detached from the cell culture flask by trypsin treatment and transfected with plasmid pFB-mAP2.2-eGFP using the Nucleofector device (Lonza) and the Amaxa Cell Line Nucleofector Kit V (Lonza) according to the manufacturer's instructions. Post-transfection, cells were seeded, cultured to confluence and exposed to adipocyte differentiation conditions 3 days post-transfection: DMEM supplemented with 10% FCS, 0.5 µM dexamethasone (Sigma-Aldrich, Taufkirchen, Germany), 0.5 µM 3-isobutyl-1-methylxanthine (IBMX) (Sigma-Aldrich), 1.67 µM insulin (Sigma-Aldrich) and 1 mM Rosiglitazone (Boehringer-Ingelheim, Ingelheim, Germany). Two days later, the induction medium was replaced by DMEM containing 10% FCS and 1.67 µM insulin for another 2 days. Thereafter, cells were incubated in DMEM with 10% FCS. At the indicated time points (Fig. S3), cells were harvested by removing the supernatant, washing twice with 1× DPBS and lyzing the cells in buffer RLT (RNeasyMini Kit; Qiagen, Hilden, Germany) containing 1% β-mercaptoethanol. Total RNA was extracted (RNeasy Mini kit; Qiagen) and cDNA was synthesized (High Capacity cDNA Reverse Transcription Kit (Applied Biosystems)). QPCR was performed followed by relative quantification of (trans-)gene (eGFP and endogenous adiponectin) and reference gene expression (RNA pol-II) using a *TaqMan HT 7900 System (Applied Biosystems*; for primer and probe sequences see S1 Table).

### Oil red O staining

Lipid droplets in mature adipocytes were stained with oil red O. Briefly, cells were fixed with 3.7% formalin for 1 hour at 37°C and washed with distilled water prior to incubation with 0.2% (w/v) filtered oil red O solution (Sigma-Aldrich) for two hours at room temperature. Subsequently, cells were washed twice with distilled water to remove excess dye and analyzed by microscopy (Zeiss Axiovert 40 CFL, Carl Zeiss, Oberkochen, Germany).

### Animal procedures

C57BL/6 mice (Charles River Laboratories, Kisslegg, Germany) had regular access to chow and water. Recombinant AAV8-CMV-eGFP, rAAV8-mAP2.2-eGFP or rAAV8-mAP2.2-PlinA were administered via tail vein injection into 8 week old female mice at a dose of 10^12^ genomic vector particles per animal diluted with 1× DPBS in a total volume of 200 µl. Injection of 1× DPBS was used as a control. Animals were sacrificed three weeks after injection and organs were isolated for further analyses. For fat pad injection 8 week old male C57BL/6 mice were injected into one epididymal fat pad with the equivalent numbers of 4.2×10^10^ vector genomes of either rAAV or rAd per fat pad. Animals were sacrificed 4 weeks post-injection. To assess metabolic parameters mice were analyzed using the Phenomaster Homecage System (TSE Systems GmbH, Bad Homburg, Germany) according to the standard protocol.

### Evaluation of transduction efficiency by qPCR

To determine transduction efficiency *in vivo*, DNA was isolated from tissue samples (DNeasy Blood&Tissue Kit, Qiagen), followed by qPCR analysis using *egfp*- or mAP2.2-promoter-specific *TaqMan* primer-probe sets (S1 Table). Subsequently, the number of vector genomes was normalized to the amount of DNA and thus calculated per ng total DNA. All reactions were performed in triplicates and analyzed using SDS2.4 software (Applied Biosystems).

### Evaluation of transcriptional activity by qPCR

Total RNA was isolated from tissue samples by phenol-chloroform extraction followed by processing of the aqueous phase with the RNeasy Mini Kit (Qiagen) according to the manufacturer's instructions. Two micrograms of total RNA were reverse transcribed using the High Capacity cDNA Reverse Transcription Kit (Applied Biosystems). cDNA corresponding to 20 ng of total RNA was subjected to qPCR analyses in order to detect expression of *egfp*, codon-optimized or endogenous *plina*. The housekeeping genes RNA pol-II or 36B4 were used as reference genes for normalization (primer and probe sequences are listed in S1 Table). All reactions were performed in triplicates and analyzed using SDS2.4 software (Applied Biosystems). Relative quantification was carried out with the standard curve method and standard curves were prepared by serial dilution of a rAAV8-CMV-treated liver cDNA sample.

### Histological fluorescence analysis

Organ explants were immediately deep-frozen in liquid nitrogen and stored at −80°C until the day of sectioning. Twelve micrometer sections of frozen tissue were prepared using a cryotome (CryoStar NX70, Thermo Scientific, Schwerte, Germany), air-dried, embedded in Aquatex (Merck, Darmstadt, Germany) and analyzed by confocal microscopy (Zeiss Axio LSM 700, Carl Zeiss). Confocal overview images with a z-dimension of 3.9 µm were obtained using the ZEN software (Carl Zeiss). To quantify eGFP fluorescence, the mean fluorescence intensity (MFI) of five random, independent fields of view per animal was analyzed using the ZEN “Histo” function.

### Serum measurements

Blood glucose was measured with a glycometer (Contour, BAYER AG, Leverkusen, Germany). Free fatty acids were measured with the NEAFA-HR kit (Wako, Neuss, Germany).

### Statistical analysis

Statistical analysis was carried out by unpaired Student's *t*-test or one-way ANOVA when comparing more than two groups using the GraphPad Prism software (Version 5.04, GraphPad Software, Inc., La Jolla, CA, USA). *P*-values<0.05 were considered statistically significant. Three gradations were used to illustrate the significance level: ***P<0.001; **P<0.01; and *P<0.05.

## Supporting Information

S1 Fig **EGFP expression in off-target organs following local injection into visceral fat pads.** C57BL/6 mice were injected with 4.2×10^10^ VG per fat pad of rAAV-CMV-eGFP vectors or rAd-CMV-eGFP, respectively. Four weeks post-injection, mice were sacrificed and eGFP expression was determined in cDNA from non-injected fat pads, subcutaneous fat, liver, spleen, small intestine and colon by qPCR analysis. Normalization to the housekeeping gene 36B4 was performed and relative gene expression is depicted in the graph. Values indicate the mean of three animals, error bars show SEM. rAAV1: white bars, rAAV2: dotted bars, rAAV5: striped bars, rAAV6: hatched bars, rAAV8: checkered bars, Adeno: black bars.
(TIF)

S2 Fig **Sequence of mAP2.2 promoter.** Sequence of the murine adiponectin promoter fragment (Genbank accession no. AF304466) [17] used in the present study.
(TIF)

S3 Fig ***In vitro*** **validation of the mAP2.2 promoter.** 3T3L1 cells were transfected with plasmid pFB-mAP2.2-eGFP using the Nucleofector system. Post transfection, cells were seeded, grown to confluence and differentiated to adipocytes (black bars) or left in an undifferentiated stadium (white bars). At 2, 4, 6, 8 and 10 days after induction of differentiation, cells were harvested, total RNA was extracted and cDNA was synthesized. QPCR was performed followed by relative quantification of eGFP (a), endogenous adiponectin (b) and reference gene expression (RNA-pol-II). Values indicate the mean normalized target/reference ratio of duplicate experiments. (c) At ten days post-differentiation, lipid droplets inside mature adipocytes were stained with oil red O. Staining was documented by light microscopy using a 40× magnification.
(TIF)

S4 Fig **Transduction efficiency (a), endogenous PlinA (b) and eGFP (c) expression following systemic application of rAAV8-mAP2.2-eGFP and rAAV8-mAP2.2-PlinA.** C57BL/6 mice were injected with 10^12^ VG per animal of rAAV vectors (rAAV8-mAP2.2-eGFP: hatched bars, rAAV8-mAP2.2-PlinA: striped bars) or PBS (white bars) as a mock control via the tail vein. Three weeks post-application, mice were sacrificed and tissue samples from subcutaneous (sc) fat, visceral (vis) fat depots and liver were taken. (a) Biodistribution of vector genomes indicating transduction efficiency was assessed by measuring mAP2.2 copies per ng total DNA in the different organs via qPCR. The mean value obtained for liver samples of the rAAV8-mAP2.2-PlinA group was set to 100%. (b) Endogenous PlinA expression levels in vis and sc fat as well as liver were determined by qPCR and normalized to RNA-pol-II. Values indicate the mean normalized target/reference ratio. (c) EGFP expression levels were determined by qPCR and normalized to the housekeeping gene RNA-pol-II. The mean of the target/reference ratio calculated for liver samples of the rAAV8-CMV-eGFP group was set to 100%. Bar charts show the mean of five animals, error bars represent SEM.
(TIF)

S1 Table **Primer and probe sequences.** All primers and probes were purchased from Sigma-Aldrich. Probes were labeled with 6-carboxyfluorescein (FAM) at the 5′-end and with tetramethylrhodamine (TAMRA) at the 3′-end of the sequence. For detection of mouse adiponectin, a primer-probe set was ordered from Life Technologies (TaqMan Gene Expression Assay for NM_009605.4, Assay ID: Mm00456425_m1).
(DOCX)

## References

[1] Barness LA, Opitz JM, Gilbert-Barness E (2007) Obesity: genetic, molecular, and environmental aspects. Am J Med Genet A 143A:3016–3034 10.1002/ajmg.a.32035 18000969

[2] Casteilla L, Cousin B, Planat-Benard V, Laharrague P, Carmona M, et al (2008) Virus-based gene transfer approaches and adipose tissue biology. Curr Gene Ther 8:79–87. 18393829 10.2174/156652308784049354

[3] Brasaemle DL (2007) Thematic review series: adipocyte biology. The perilipin family of structural lipid droplet proteins: stabilization of lipid droplets and control of lipolysis. J Lipid Res 48:2547–2559 10.1194/jlr.R700014-JLR200 17878492

[4] Seale P, Conroe HM, Estall J, Kajimura S, Frontini A, et al (2011) Prdm16 determines the thermogenic program of subcutaneous white adipose tissue in mice. J Clin Invest 121:96–105 10.1172/JCI44271 21123942 PMC3007155

[5] Enerbäck S (2009) The origins of brown adipose tissue. N Engl J Med 360:2021–2023 10.1056/NEJMcibr0809610 19420373

[6] Ishibashi J, Seale P (2010) Medicine. Beige can be slimming. Science 328:1113–1114 10.1126/science.1190816 20448151 PMC2907667

[7] Cristancho AG, Lazar MA (2011) Forming functional fat: a growing understanding of adipocyte differentiation. Nat Rev Mol Cell Biol 12:722–734 10.1038/nrm3198 21952300 PMC7171550

[8] Jimenez V, Munoz S, Casana E, Mallol C, Elias I, et al (2013) In Vivo Adeno-Associated Viral Vector-Mediated Genetic Engineering of White and Brown Adipose Tissue in Adult Mice. Diabetes 62:4012–4022 10.2337/db13-0311 24043756 PMC3837045

[9] O'Neill SM, Hinkle C, Chen S-J, Sandhu A, Hovhannisyan R, et al (2014) Targeting adipose tissue via systemic gene therapy. Gene Ther 21:653–61 10.1038/gt.2014.38 24830434 PMC4342115

[10] Büning H, Perabo L, Coutelle O, Quadt-Humme S, Hallek M (2008) Recent developments in adeno-associated virus vector technology. J Gene Med 10:717–733 10.1002/jgm.1205 18452237

[11] Halder S, Ng R, Agbandje-McKenna M (2012) Parvoviruses: structure and infection. Future Virol 7:253–278 10.2217/fvl.12.12

[12] Asokan A, Schaffer DV, Jude Samulski R (2012) The AAV Vector Toolkit: Poised at the Clinical Crossroads. Mol Ther 20:699–708 10.1038/mt.2011.287 22273577 PMC3321598

[13] Mizukami H, Mimuro J, Ogura T, Okada T, Urabe M, et al (2006) Adipose tissue as a novel target for in vivo gene transfer by adeno-associated viral vectors. Hum Gene Ther 17:921–928 10.1089/hum.2006.17.921 16972760

[14] Rabinowitz JE, Rolling F, Li C, Conrath H, Xiao W, et al (2002) Cross-packaging of a single adeno-associated virus (AAV) type 2 vector genome into multiple AAV serotypes enables transduction with broad specificity. J Virol 76:791–801. 11752169 10.1128/JVI.76.2.791-801.2002 PMC136844

[15] Mingozzi F, High KA (2011) Therapeutic in vivo gene transfer for genetic disease using AAV: progress and challenges. Nat Rev Genet 12:341–355 10.1038/nrg2988 21499295

[16] Zincarelli C, Soltys S, Rengo G, Rabinowitz JE (2008) Analysis of AAV Serotypes 1–9 Mediated Gene Expression and Tropism in Mice After Systemic Injection. Mol Ther 16:1073–1080 10.1038/mt.2008.76 18414476

[17] Koshiishi C, Park H-M, Uchiyama H, Tanaka Y (2008) Regulation of expression of the mouse adiponectin gene by the C/EBP family via a novel enhancer region. Gene 424:141–146 10.1016/j.gene.2008.07.039 18760339

[18] Srivastava A, Lusby EW, Berns KI (1983) Nucleotide sequence and organization of the adeno-associated virus 2 genome. J Virol 45:555–564. 6300419 10.1128/jvi.45.2.555-564.1983 PMC256449

[19] Asokan A, Conway JC, Phillips JL, Li C, Hegge J, et al (2010) Reengineering a receptor footprint of adeno-associated virus enables selective and systemic gene transfer to muscle. Nat Biotechnol 28:79–82 10.1038/nbt.1599 20037580 PMC2912150

[20] Schievenbusch S, Strack I, Scheffler M, Nischt R, Coutelle O, et al (2010) Combined paracrine and endocrine AAV9 mediated expression of hepatocyte growth factor for the treatment of renal fibrosis. Mol Ther J Am Soc Gene Ther 18:1302–1309 10.1038/mt.2010.71 PMC2911253 20424598

[21] Tansey JT, Sztalryd C, Gruia-Gray J, Roush DL, Zee JV, et al (2001) Perilipin ablation results in a lean mouse with aberrant adipocyte lipolysis, enhanced leptin production, and resistance to diet-induced obesity. Proc Natl Acad Sci U S A 98:6494–6499 10.1073/pnas.101042998 11371650 PMC33496

[22] Nedergaard J, Bengtsson T, Cannon B (2007) Unexpected evidence for active brown adipose tissue in adult humans. Am J Physiol Endocrinol Metab 293:E444–E452 10.1152/ajpendo.00691.2006 17473055

[23] Petrovic N, Walden TB, Shabalina IG, Timmons JA, Cannon B, et al (2009) Chronic Peroxisome Proliferator-activated Receptor (PPAR) Activation of Epididymally Derived White Adipocyte Cultures Reveals a Population of Thermogenically Competent, UCP1-containing Adipocytes Molecularly Distinct from Classic Brown Adipocytes. J Biol Chem 285:7153–7164 10.1074/jbc.M109.053942 20028987 PMC2844165

[24] Nguyen T, Lau DCW (2012) The Obesity Epidemic and Its Impact on Hypertension. Can J Cardiol 28:326–333 10.1016/j.cjca.2012.01.001 22595448

[25] Richardson VR, Smith KA, Carter AM (2013) Adipose tissue inflammation: Feeding the development of type 2 diabetes mellitus. Immunobiology. 10.1016/j.imbio.2013.05.002 23816302

[26] Rosenwald M, Perdikari A, Rülicke T, Wolfrum C (2013) Bi-directional interconversion of brite and white adipocytes. Nat Cell Biol 15:659–667 10.1038/ncb2740 23624403

[27] Coughlan L, Alba R, Parker AL, Bradshaw AC, McNeish IA, et al (2010) Tropism-Modification Strategies for Targeted Gene Delivery Using Adenoviral Vectors. Viruses 2:2290–2355 10.3390/v2102290 21994621 PMC3185574

[28] Schagen FHE, Ossevoort M, Toes REM, Hoeben RC (2004) Immune responses against adenoviral vectors and their transgene products: a review of strategies for evasion. Crit Rev Oncol Hematol 50:51–70 10.1016/S1040-8428(03)00172-0 15094159

[29] Liu Q, Muruve DA (2003) Molecular basis of the inflammatory response to adenovirus vectors. Gene Ther 10:935–940 10.1038/sj.gt.3302036 12756413

[30] Rauschhuber C, Noske N, Ehrhardt A (2012) New insights into stability of recombinant adenovirus vector genomes in mammalian cells. Eur J Cell Biol 91:2–9 10.1016/j.ejcb.2011.01.006 21440326

[31] Martinez-Botas J, Anderson JB, Tessier D, Lapillonne A, Chang BH, et al (2000) Absence of perilipin results in leanness and reverses obesity in Lepr(db/db) mice. Nat Genet 26:474–479 10.1038/82630 11101849

[32] Brown BD, Naldini L (2009) Exploiting and antagonizing microRNA regulation for therapeutic and experimental applications. Nat Rev Genet 10:578–585 10.1038/nrg2628 19609263

[33] Geisler A, Jungmann A, Kurreck J, Poller W, Katus HA, et al (2010) microRNA122-regulated transgene expression increases specificity of cardiac gene transfer upon intravenous delivery of AAV9 vectors. Gene Ther 18:199–209 10.1038/gt.2010.141 21048795

[34] Xie J, Xie Q, Zhang H, Ameres SL, Hung J-H, et al (2011) MicroRNA-regulated, Systemically Delivered rAAV9: A Step Closer to CNS-restricted Transgene Expression. Mol Ther 19:526–535 10.1038/mt.2010.279 21179009 PMC3048189

[35] Grimm D, Lee JS, Wang L, Desai T, Akache B, et al (2008) In vitro and in vivo gene therapy vector evolution via multispecies interbreeding and retargeting of adeno-associated viruses. J Virol 82:5887–5911 10.1128/JVI.00254-08 18400866 PMC2395137

[36] Raupp C, Naumer M, Muller OJ, Gurda BL, Agbandje-McKenna M, et al (2012) The threefold protrusions of AAV8 are involved in cell surface targeting as well as post attachment processing. J Virol 86:9396–9408 10.1128/JVI.00209-12 22718833 PMC3416165

[37] Urabe M, Ding C, Kotin RM (2002) Insect cells as a factory to produce adeno-associated virus type 2 vectors. Hum Gene Ther 13:1935–1943 10.1089/10430340260355347 12427305

[38] Moullier P, Snyder RO (2008) International Efforts for Recombinant Adeno-associated Viral Vector Reference Standards. Mol Ther 16:1185–1188 10.1038/mt.2008.125 18574495

[39] Grimm D (2003) Helper virus-free, optically controllable, and two-plasmid-based production of adeno-associated virus vectors of serotypes 1 to 6. Mol Ther 7:839–850 10.1016/S1525-0016(03)00095-9 12788658

