# Supplementary figures and images for: Gene Delivery to Adipose Tissue Using Transcriptionally Targeted rAAV8 Vectors
Source: PLoS One. 2014 Dec 31;9(12):e116288. doi: 10.1371/journal.pone.0116288 (PMC4281237; doi:10.1371/journal.pone.0116288)

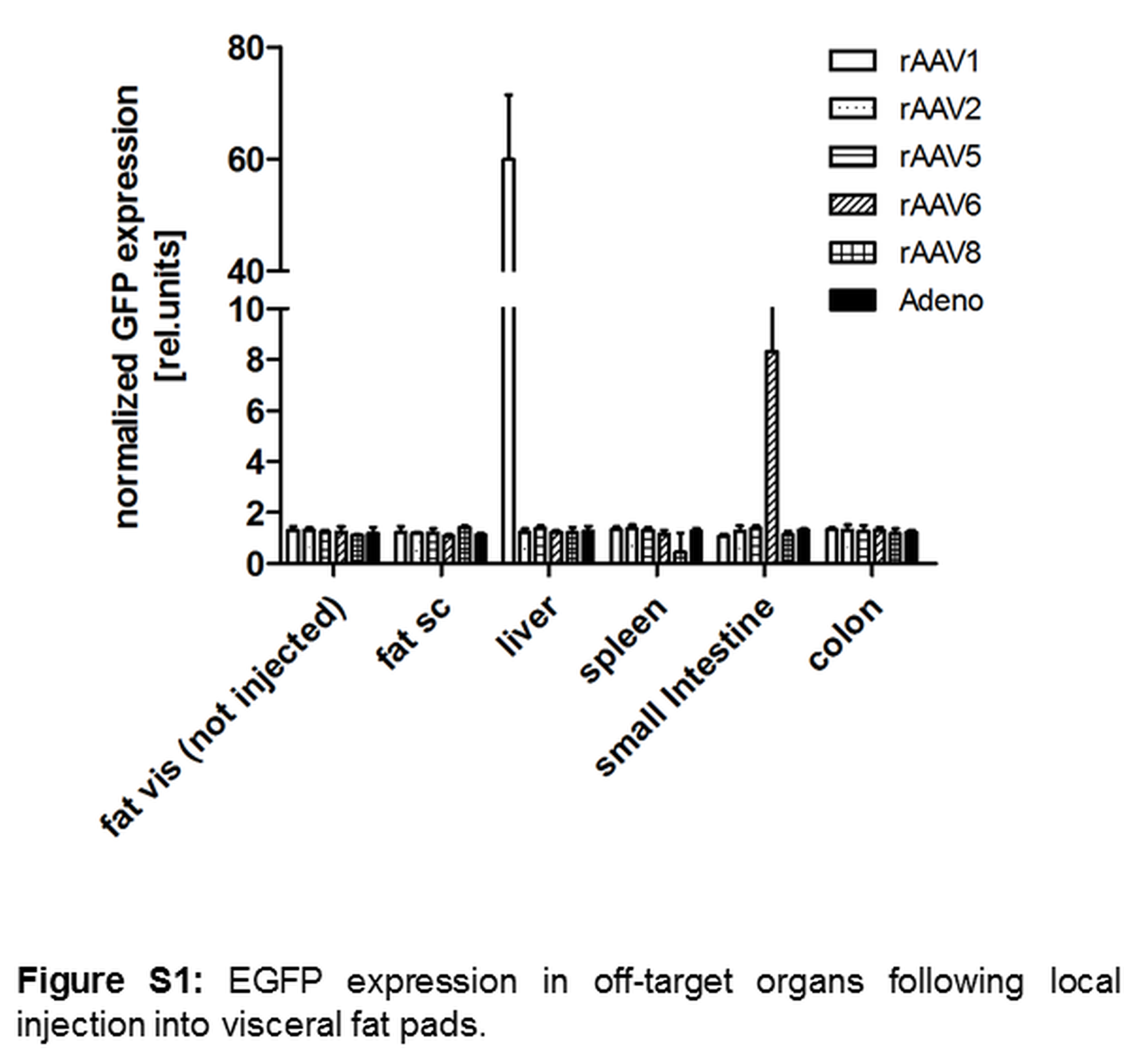

Supplement: S1 Fig — EGFP expression in off-target organs following local injection into visceral fat pads. C57BL/6 mice were injected with 4.2×10^10^ VG per fat pad of rAAV-CMV-eGFP vectors or rAd-CMV-eGFP, respectively. Four weeks post-injection, mice were sacrificed and eGFP expression was determined in cDNA from non-injected fat pads, subcutaneous fat, liver, spleen, small intestine and colon by qPCR analysis. Normalization to the housekeeping gene 36B4 was performed and relative gene expression is depicted in the graph. Values indicate the mean of three animals, error bars show SEM. rAAV1: white bars, rAAV2: dotted bars, rAAV5: striped bars, rAAV6: hatched bars, rAAV8: checkered bars, Adeno: black bars. (TIF) [file pone.0116288.s001.tif]

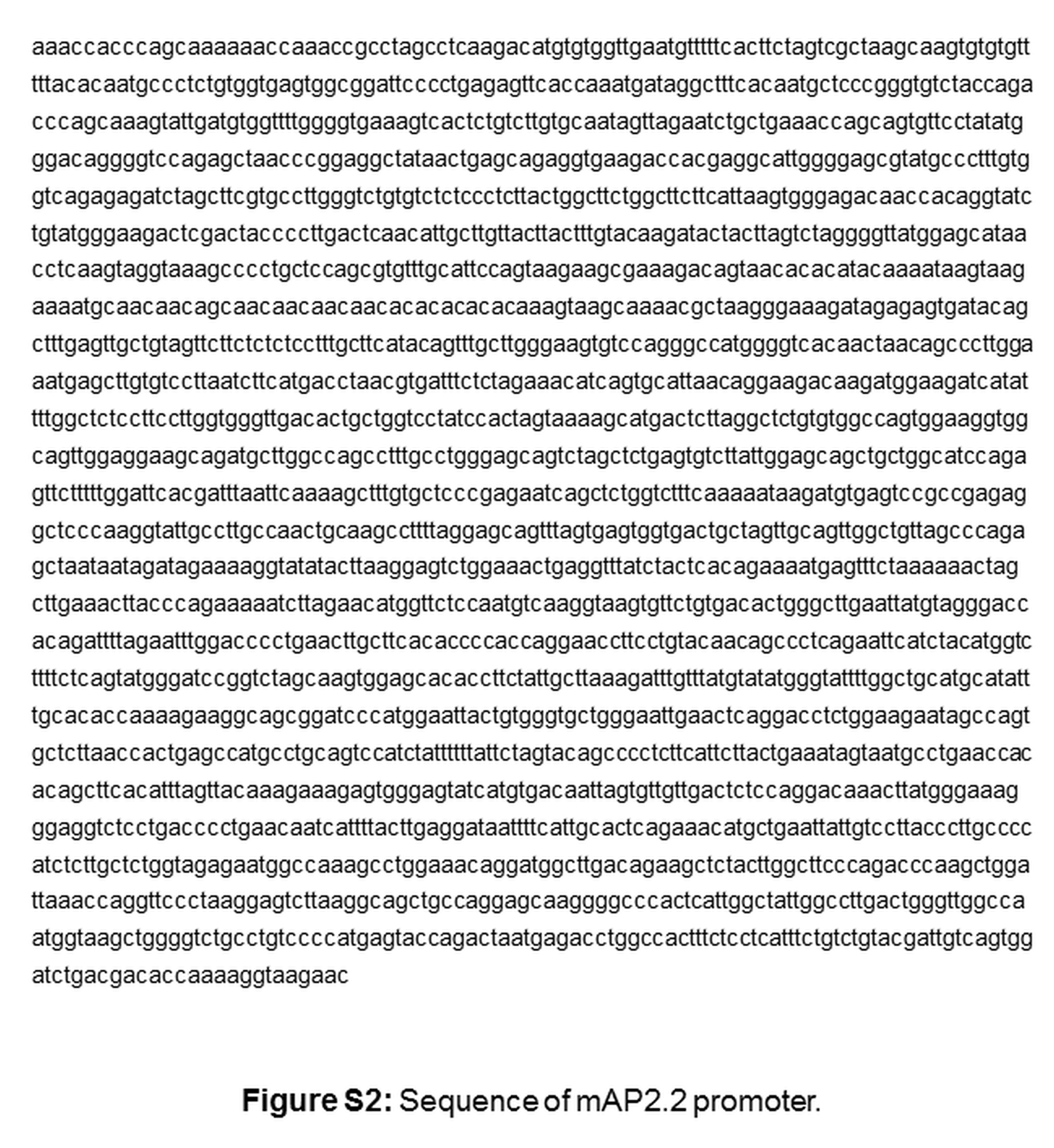

Supplement: S2 Fig — Sequence of mAP2.2 promoter. Sequence of the murine adiponectin promoter fragment (Genbank accession no. AF304466) [17] used in the present study. (TIF) [file pone.0116288.s002.tif]

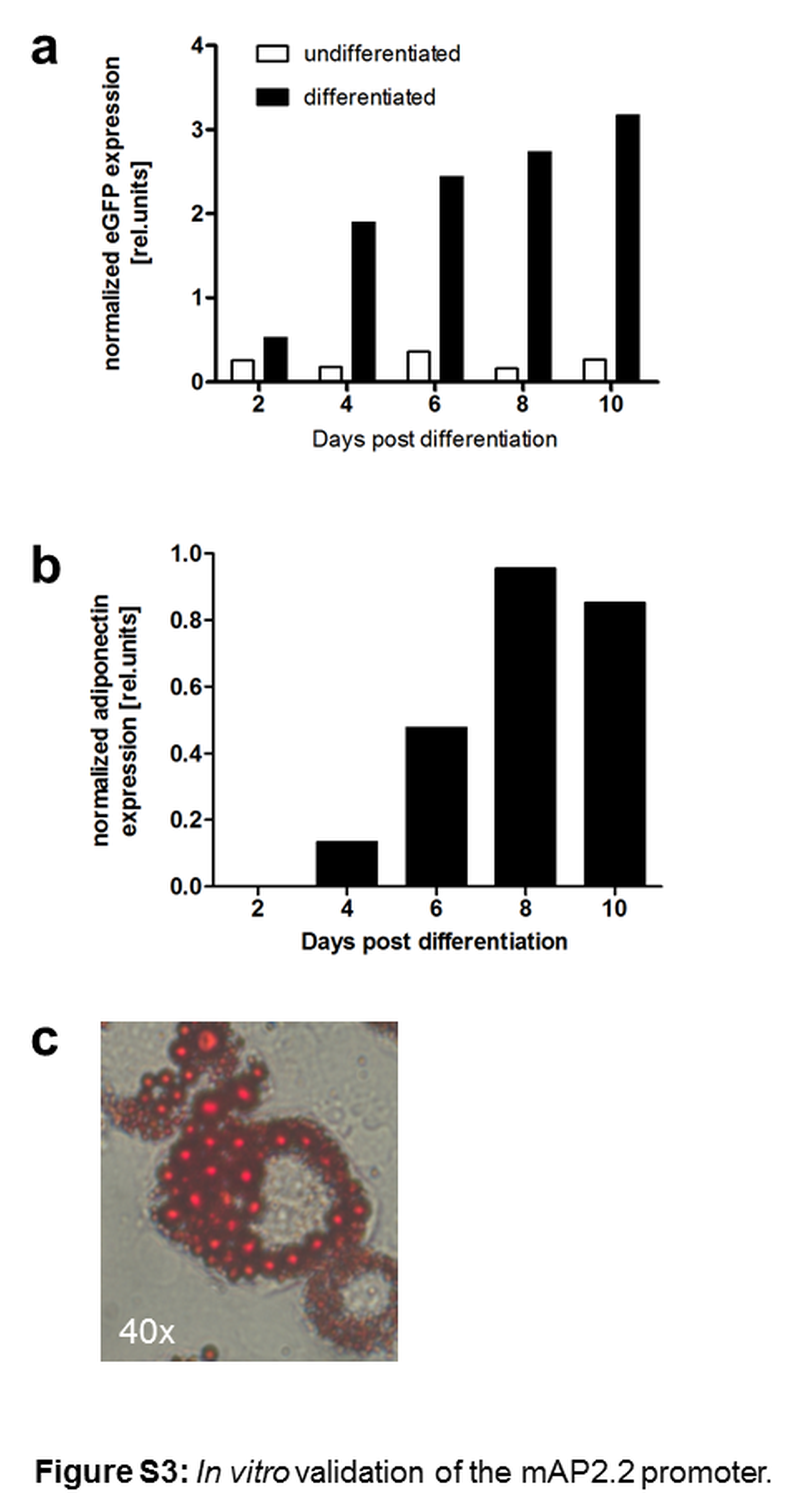

Supplement: S3 Fig — In vitro validation of the mAP2.2 promoter. 3T3L1 cells were transfected with plasmid pFB-mAP2.2-eGFP using the Nucleofector system. Post transfection, cells were seeded, grown to confluence and differentiated to adipocytes (black bars) or left in an undifferentiated stadium (white bars). At 2, 4, 6, 8 and 10 days after induction of differentiation, cells were harvested, total RNA was extracted and cDNA was synthesized. QPCR was performed followed by relative quantification of eGFP (a), endogenous adiponectin (b) and reference gene expression (RNA-pol-II). Values indicate the mean normalized target/reference ratio of duplicate experiments. (c) At ten days post-differentiation, lipid droplets inside mature adipocytes were stained with oil red O. Staining was documented by light microscopy using a 40× magnification. (TIF) [file pone.0116288.s003.tif]

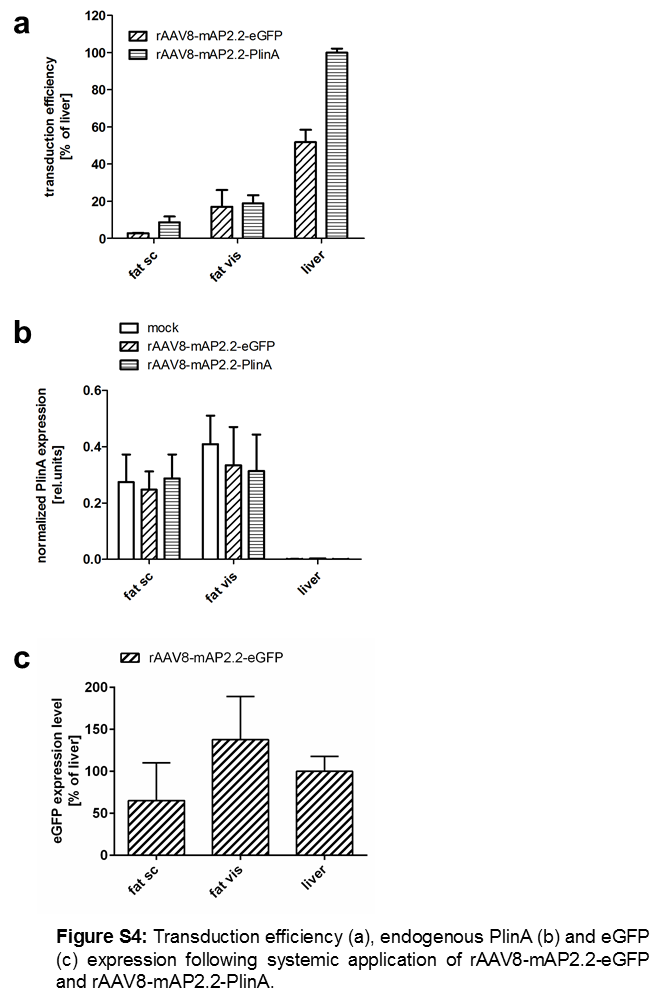

Supplement: S4 Fig — Transduction efficiency (a), endogenous PlinA (b) and eGFP (c) expression following systemic application of rAAV8-mAP2.2-eGFP and rAAV8-mAP2.2-PlinA. C57BL/6 mice were injected with 1012 VG per animal of rAAV vectors (rAAV8-mAP2.2-eGFP: hatched bars, rAAV8-mAP2.2-PlinA: striped bars) or PBS (white bars) as a mock control via the tail vein. Three weeks post-application, mice were sacrificed and tissue samples from subcutaneous (sc) fat, visceral (vis) fat depots and liver were taken. (a) Biodistribution of vector genomes indicating transduction efficiency was assessed by measuring mAP2.2 copies per ng total DNA in the different organs via qPCR. The mean value obtained for liver samples of the rAAV8-mAP2.2-PlinA group was set to 100%. (b) Endogenous PlinA expression levels in vis and sc fat as well as liver were determined by qPCR and normalized to RNA-pol-II. Values indicate the mean normalized target/reference ratio. (c) EGFP expression levels were determined by qPCR and normalized to the housekeeping gene RNA-pol-II. The mean of the target/reference ratio calculated for liver samples of the rAAV8-CMV-eGFP group was set to 100%. Bar charts show the mean of five animals, error bars represent SEM. (TIF) [file pone.0116288.s004.tif]
