# Supplementary material for: Gene Delivery to Adipose Tissue Using Transcriptionally Targeted rAAV8 Vectors
Source: PLoS One. 2014 Dec 31;9(12):e116288. doi: 10.1371/journal.pone.0116288 (PMC4281237; doi:10.1371/journal.pone.0116288)
Supplement: S1 Table — Primer and probe sequences. All primers and probes were purchased from Sigma-Aldrich. Probes were labeled with 6-carboxyfluorescein (FAM) at the 5′-end and with tetramethylrhodamine (TAMRA) at the 3′-end of the sequence. For detection of mouse adiponectin, a primer-probe set was ordered from Life Technologies (TaqMan Gene Expression Assay for NM_009605.4, Assay ID: Mm00456425_m1). (DOCX) [file pone.0116288.s005.docx]

| **Primer / probe name** | **Sequence (5‘- 3‘)** |
| --- | --- |
| eGFP_fw | CTGCTGCCCGACAACCA |
| eGFP_rev | TGTGATCGCGCTTCTCGTT |
| eGFP_probe | TACCTGAGCACCCAGTCCGCCCT |
| mAP2.2-Prom_fw | CCTTGCCCCATCTCTTGCT |
| mAP2.2-Prom _rev | AGAGCTTCTGTCAAGCCATCCT |
| mAP2.2-Prom _probe | TGGTAGAGAATGGCCAAAGCCTGGAA |
| PlinA_codonopt_fw | GTGCTTCCAGAAAACCTACAACAG |
| PlinA_codonopt_rev | CTCGTAAGCGTTGCACACAGA |
| PlinA_codonopt_probe | ACCAAAGAGGCCCACCCCCTGG |
| PlinA_endo_fw | TCGAAGCGCCAGGAACAG |
| PlinA_endo_rev | GGCCCCCAGAACCTTGTC |
| PlinA_endo_probe | ATCAGTGTGCCCATTGCAAGCACCT |
| RNA-pol-II_fw | GCCAAAGACTCCTTCACTCACTGT |
| RNA-pol-II_rev | TTCCAAGCGGCAAAGAATGT |
| RNA-pol-II_probe | TGGCTCTTTCAGCATCTCGTGCAGATT |
| 36B4_fw | GCCGTGATGCCCAGGGAAGA |
| 36B4_rev | CATCTGCTTGGAGCCCACGTT |

**Table S1: Primer and probe sequences.**
